# The Draft Genome Sequence of *Burkholderia* sp. Strain Nafp2/4-1b Confirms Its Ability To Produce the Plant Growth-Promoting Traits Pyoverdine Siderophores, 1-Aminocyclopropane-1-Carboxylate Deaminase, and the Phytohormone Auxin

**DOI:** 10.1128/MRA.01383-18

**Published:** 2018-12-13

**Authors:** Ahmed Idris Hassen, L. S. Khambani, Z. H. Swanevelder

**Affiliations:** a Agricultural Research Council, Plant Health and Protection, Queenswood, Pretoria, South Africa; b Agricultural Research Council, Biotechnology Platform, Onderstpoort, South Africa; Queens College

## Abstract

*Burkholderia* sp. strain Nafp2/4-1b is a rhizobacterium isolated from the rhizosphere of grassland in South Africa. This draft genome report confirms the presence of genes related to iron acquisition, alleviation of abiotic stress in plants, and other essential traits of plant growth-promoting rhizobacteria (PGPR) that signify the potential of this strain as a plant growth-promoting agent.

## Abstract (precis)

*Burkholderia* sp. strain Nafp2/4-1b is a rhizobacterium isolated from the rhizosphere of grassland in South Africa.

## ANNOUNCEMENT

Members of the genus *Burkholderia* are Gram-negative bacteria belonging to the beta subclass of *Proteobacteria* and are part of a larger group of rhizobacteria that can establish a positive interaction within plant roots. Several strains within the genus are known to colonize the rhizosphere and play key roles in crop yield, plant health, and soil fertility (1, 2). They are therefore often referred to as plant growth-promoting rhizobacteria (PGPR) due to their ability to improve the growth and health of several economically important crops (3, 4).

The *Burkholderia* sp. strain presented here, Nafp2/4-1b, was initially isolated from the rhizosphere of grassland in South Africa. For isolation, a 100-µl aliquot of serially diluted rhizosphere soil suspension was plated on solid nutrient agar (NA) medium and was incubated at 28°C for 24 h. Selected pure colonies of the bacteria were evaluated for their growth-promoting activity in maize (*Zea mays* L.) and *in vitro* detection of major PGPR traits.

Based on the growth-promoting traits exhibited in both the gnotobiotic test and the *in vitro* characterization, the genome of this strain was sequenced to confirm and increase our understanding of its PGPR properties for possible use as a biofertilizer inoculant in crop production. For DNA extraction, a single pure colony of the bacterium was grown in Luria Bertani (LB) broth for 24 h on a rotary shaker at 28°C. One milliliter of the culture suspension was used to extract total DNA using the Wizard genomic DNA purification kit and protocol (Promega, Madison, WI, USA). Paired-end DNA libraries were prepared using the Nextera protocol (Illumina, USA) for sequencing on a HiSeq 2500 instrument (Illumina, USA) at the Agricultural Research Council’s Biotechnology Platform in Onderstpoort, South Africa. About 13,542,806 paired-end sequences (125 bp each) were obtained prior to adaptor quality trimming and the merging of overlapped reads. CLC Genomics Workbench 8.5.1 was used for the trimming and genome assembly, with the default setting of 20 bp used. This resulted in a final number of merged and unmerged reads of 8,610,493 being used to produce the final assembly. The draft genome consists of 92 scaffolds (i.e., 110 contigs), with an *N*_50_ value of 322,407 bp and a maximum scaffold size of 1,302,675 bp. The total draft genome size is estimated at 7,786,570 bp (i.e., 123× coverage), with a GC content of 66.2%. The NCBI Prokaryotic Genome Annotation Pipeline (PGAP) was used for genome annotation (5, 6), which identified 7,223 putative coding regions.

This draft genome sequence of *Burkholderia* sp. Nafp2/4-1b revealed the presence of the genes that code for the following PGPR traits: 1**-**aminocyclopropane-1-carboxylate (ACC) deaminase (contig 3), iron-binding pyoverdine siderophore biosynthesis (contig 28), tryptophan synthase beta and alpha chains involved in auxin biosynthesis (contig 5), ferric (Fe^+3^) hydroxamate siderophore iron transporter (*FhuB* gene) (contig 28), and ATP binding cassette (ABC) transporters that mediate the uptake of the heavy metal-complexing peptides iron, siderophores, and phytochelatin (contig 1).

### Data availability.

This whole-genome shotgun project has been deposited at DDBJ/ENA/GenBank under the accession number PXXL00000000. The version described in this paper is version PXXL01000000. The SRA accession number for the raw data is PRJNA438206.
